# Fecal Carriage of Colibactin-Encoding *Escherichia coli* Associated With Colorectal Cancer Among a Student Populace

**DOI:** 10.1093/ofid/ofae106

**Published:** 2024-02-26

**Authors:** Paul A Akinduti, Ovbiosa O Izevbigie, Omobolanle A Akinduti, Ezekiel O Enwose, Emmanuel O Amoo

**Affiliations:** Microbiology Unit, Department of Biological Sciences, Covenant University, Ota, Nigeria; Microbiology Unit, Department of Biological Sciences, Covenant University, Ota, Nigeria; Department of Nursing, Federal Medical Centre, Abeokuta, Nigeria; Department of Medical Laboratory Sciences, Neuropsychiatric Hospital, Aro Abeokuta, Nigeria; Demography and Social Statistics, Covenant University, Ota, Nigeria

**Keywords:** antibiotics, colibactin, colorectal cancer, *Escherichia coli*, fecal

## Abstract

Fecal carriage of the colibactin (*clb*) gene in *Escherichia coli* is described as a source that could promote carcinogenesis, progressing to colorectal cancer. The present study investigated the demographic, dietary, and antibiotic consumption variables as correlates for fecal carriage of *clb*+/*E coli* among the student populace. In a randomized cross-sectional survey, *E coli* (N = 136) from the fecal samples of eligible students were characterized and evaluated for antibiotic resistance, β-lactamase (blm), biofilm, virulence factor production, and strain tryptophan reverse mutagenic activity. The encoded *clb*+/*E coli* were analyzed for correlates with principal component analysis. Of all the *E coli* strains, a low rate of 2 *clb+/E coli* (1.5%) and higher rates of biofilm (13.2%) and blm producers (11.8%) were recorded among the mutant strains as compared with the nonmutant types. All the *clb+*/*E coli* showed complete resistance to amoxicillin, Augmentin (amoxicillin and clavulanate), gentamicin, and trimethoprim/sulfamethoxazole. The fecal *clb*-encoded *E coli* (1.5%) were not associated with demographic status, fiber-based food (odds ratio [OR], 1.03; 95% CI, 56.74–138.7; *P* = .213), alcohol (OR, 1.27; 95% CI, 61.74–147.1; *P* = .221), antibiotic consumptions (OR, 1.11; 95% CI, 61.29–145.3; *P* = .222), and handwashing (OR, 1.17; 95% CI, 60.19–145.5; *P* = .216). The hierarchical cluster of blm+/*E coli* revealed high-level resistance with a multiantibiotic resistance index ≥0.2 (*P* < .05). Only 12% of all strains were tryptophan mutant/*blm*+, and 1.5% of *clb*+/EC^blm+^ were observed in fecal samples with a 452–base pair size. Trimethoprim/sulfamethoxazole and biofilm production positively regressed with *clb* expression (*P* > .05). Principal component analysis score plot indicated an association of *clb*+/EC^blm+^ with dietary pattern, alcohol, blm, and hemolysin production. The combined activity of blm and biofilm production in the gut microbiota could promote *clb*+/*E coli* colonization, facilitating genotoxin production and possible colorectal cancer induction.

## INTRODUCTION

According to the World Health Organization, colorectal cancer (CRC) is identified as one of the leading causes of death [1] and is mostly diagnosed in men as compared with women [1, 2]. In Europe, the early onset of CRC incidence was reported among individuals aged 20 to 39 years, of which diet and lifestyle play a significant role [2, 3]. In the recent past, the CRC incidence rate is getting higher in developing countries with the burden of infectious diseases, and this further predisposes several people to the risk of CRC [4, 5]. Genetic inheritance cannot be ruled out as a significant risk for CRC, while several reports have indicated the following to be associated with the incidence of CRC among the African populace: alcohol, smoking, chemical condiments as food spice (organic or inorganic type), bacteria, oncogenic viruses, diet, hormonal imbalance, and environmental pollution [6–8]. To date, the association of the dietary pattern and lifestyle of students with CRC is yet to be confirmed, but plant-based diets and few dairy products are reported to lower the risk of CRC while diets rich in meats, refined grains, and sugar tend to increase gut susceptibility [9]. The association between CRC and alcohol remains unclear.

Antibiotic use was identified to possibly increase CRC risk by altering the profile of the gut microbiota [10]. The persistent misuse of antibiotics for the treatment of gastrointestinal diseases is suggestive of the functional role of different antibiotic classes (quinolones and sulfonamides and/or trimethoprims) to increase the gut’s chances for proximal colon cancer and possible specific colorectal carcinogenesis [11]. Hygiene practices with regular handwashing after use of the toilet and before meals were significantly associated with the development of CRC in a survey conducted among the Pakistani population [12]. In most communities in developing countries with open defecation and poor hand hygiene after use of the toilet [13], there is possible transmission of fecal pathogenic *Escherichia coli* causing severe intestinal morbidity.

Globally, there is limited information on the association of β-lactamase resistance enzymes with CRC, but there is a need to investigate the contribution of β-lactamase production in *E coli* and the induction of CRC. Fecal carriage of β-lactamase–producing *E coli* demonstrating multidrug resistance poses a serious challenge to therapeutic options for colon cancer management and could enhance the severity of CRC in many individuals [14]. Among the gut microbiomes, *E coli* is commonly found as normal flora in patients with CRC and healthy individuals; however, few pathotypes were reported to induce specific cytopathic changes (megalocytosis) in the human gut via the production of toxins [15]. Colibactin is an important genotoxin produced from a 54-kb pks island, which consists of a clbA-S gene cluster encoding nonribosomal peptides and polyketide synthases with other accessory enzymes [12]. Colibactin is predominantly found in the pks genomic island, mostly reported in the *E coli* B2 phylogenetic group [15], and is associated with cytopathic changes that enhance the progression of CRC [12]. Mutant *E coli* were identified to be associated with CRC, showing invasive colonization of the lumen epithelium and producing different toxins called *cyclomodulins* (eg, colibactin, cytolethal distending toxin, cycle inhibiting factor, and cytotoxic necrotizing factor), as previously reported in patients with CRC [15, 16].

Among the students, disparities in dietary patterns, misuse of antibiotics, and hygiene status have not been studied as major factors that facilitate fecal carriage of colibactin-encoded *E coli*. The present study aims to investigate the demography and dietary pattern, biofilm, β-lactamase, and virulence factor production as correlates for fecal carriage of *clb*-encoded *E coli* as potential agents for CRC among the student populace.

## METHODS

### Study Population

This is a randomized cross-sectional study of fecal carriage of *clb*-encoded *E coli* among the student population, including undergraduate and postgraduate students. The inclusion criteria are based on their consent to participate and their status as full-time undergraduate and postgraduate students. The study excluded pregnant students, any students on medication, and students who were acutely ill or had known diseases. From the estimation of >12 000 students, the sample size (N = 136) was determined by the Fischer formula (n = *z*^2^*pq*/*d*^2^) [17]. A prevalence of the colibactin gene among the tertiary students could not be found, but the prevalence rate in a previous study was used to derive the sample size [18].

### Ethical Permission

The study was approved by the Covenant Health Research Committee, Covenant University, Ota, Nigeria (protocol NHREC/CU-HREC/11/04/2023). The informed consent of recruited individuals to participate in the study was integrated into the questionnaire, which was structured to include select demography data, feeding patterns, antibiotic use, alcohol consumption, condiment use, and hand hygiene patterns.

### Sampling and Fecal Collection

A random sampling for the fecal sample collection from the student populace was based on the exclusion criteria for the selection of respondents. Structured questionnaires were administered to obtain data on age, gender, feeding pattern and dietary practices, antibiotic use, alcohol consumption, and hygiene practices. A separate code number was given to each respondent’s questionnaire, as well as to each respective fecal sample.

### Isolation and Biotyping

Collected fecal samples (N = 136) were homogenized in sterile normal saline and plated on MacConkey lactose agar (Oxoid) for selective isolation of gram-negative bacteria and further differentiation for lactose fermentation. The plates were incubated at 37 °C for 24 hours. Based on the colonial and cellular morphology, suspected *E coli* colonies that showed lactose fermentation (pink coloration) and were slightly raised and glossy were subcultured onto eosin methylene blue agar. After incubation at 37 °C overnight as previously described [19], colonies showing phenotypic colonial appearance of differential metallic sheen were selected. Pure colonies were cultured on 7% sheep blood agar and incubated at 37 °C overnight to evaluate their hemolytic reaction. Each strain was biotyped by emulsifying a loopful of a pure colony in sterile phosphate-buffered saline and added to an API 20E panel of biochemical tests (BioMérieux) for biochemical characterization.

### Antibiotic Resistance and β-Lactamase Phenotyping

The susceptibility pattern of each characterized strain to selected antibiotics was based on the report of commonly listed antibiotics from the questionnaires obtained from the respondents. In line with the recommendation and guideline of the Clinical and Laboratory Standards Institute [20], the Kirby-Bauer disc diffusion method [21] was used to evaluate the susceptibility pattern of *E coli* to different classes of antibiotics [22]. Briefly, overnight 0.5 MacFarland turbid broth suspensions were spread smoothly onto Mueller-Hinton agar with a sterile swab stick. The following antibiotic discs were gently placed on the plate and incubated at 37 °C for 24 hours: cefprozil (10 µg), ofloxacin (30 µg), Augmentin (30 µg), nitrofurantoin (30 µg), ciprofloxacin (30 µg), ceftazidime (30 µg), gentamicin (10 µg), and ampicillin (30 µg). *E coli* strain ATCC 25922, serving as a control strain, was used as a reference. The antibiogram results were used to classify the isolates as resistant, intermediate resistant, and susceptible according to guidelines of the Clinical and Laboratory Standards Institute [20]. All isolates showing resistance to ≥3 classes of antibiotics were classified as multidrug-resistant strains. The multiantibiotic resistance index of each strain to antibiotics was also determined [23, 24]. The select multidrug-resistant strains were tested for β-lactamase production for definitive assessment of enzyme inactivation as a resistance mechanism with the starch iodide acidimetric method as previously described [25, 26] and further identified by the modified method of Stokes and Ridgway [27].

### Biofilm and Virulence Factor Detection

The ability of the multidrug-resistant strains to produce biofilm was detected by microtiter plate bioassay [28], and the level of biofilm produced was quantitatively measured by an enzyme-linked immunosorbent assay spectrophotometric reader as previously described [29]. Briefly, from overnight broth culture, 1:40 diluted broth in tryptic soy broth containing 0.25% glucose was prepared, and 200 µL from the diluted broth was inoculated in a sterile microtiter well and then incubated at 37 °C for 24 hours for biofilm production. After incubation, the medium was removed and washed twice with 0.2 mL of phosphate-buffered saline (pH 7.4) and stained with 0.1% crystal violet solution for 30 minutes. The color intensity produced after the addition of 95% ethanol was measured at 560 nm by the enzyme-linked immunosorbent assay plate reader. The absorbance of the test was compared with the negative control to determine the level of biofilm produced. Production of hemolysin was demonstrated on 5% defibrinated sheep blood agar overlaid with the Nutrient Agar base (Oxoid) after streaking a single colony and incubating the plates at 37 °C for 72 hours [30]. Detection of lipase production was performed on a tributyrin agar plate as described [30]. A single colony was cultured on the tributyrin agar plate and incubated at 37 °C for 24 hours. The observed zone of hydrolysis around the colony indicates lipase production [31]. The phenotypic assay for protease production was carried out according to Suganthi et al [32]. Briefly, 100 µL of 0.5 MacFarland turbid broth was gently placed on skim milk agar supplemented with 1% casein and allowed to be adsorbed, followed by incubation at 37 °C for 24 hours. A clear zone around the inoculum spot indicates positive casein hydrolysis.

### Strain Mutagenicity and clb Genotyping

An Ames test was used to describe the *E coli* tryptophan (trp) reverse mutagenic activity via the blocking of trp biosynthesis prior to the production of anthranilic acid as described by Arun Nagendran [33]. Overnight broth culture of *E coli* was mixed with 0.05 mg of trp in a sterile test tube that served as a test (trp+ *E coli*) and a control tube containing *E coli* mixed with normal saline (trp– *E coli*). Both tubes were incubated at 37 °C for 20 minutes. Five milliliters of the trp+ *E coli* and trp– *E coli* suspension were spread on separate agar-agar plates and incubated at 37 °C for 48 hours. After 48 hours, the number of colonies in each plate was counted. The mutagenicity of trp is proportional to the number of colonies observed. If there is a large number of colonies on the test plate in comparison with the control, then the isolates were suspected mutant strains. The carriage of clbA was determined by genotyping as described by McCarthy et al [34]. clbA is an important gene cluster in colibactin synthesis and noted as a phosphopantetheinyl transferase–encoded gene, which is majorly required for the biosynthesis and maturation of colibactin and usually associated with pathogenicity and cancer [34]. Extracted chromosomal DNA template from overnight pure broth culture was obtained with a commercial kit following the manufacturer's instruction. Polymerase chain reaction was carried out in a total volume of 20 µL containing 12 µL of 10 × Master Mix buffer, 1.0 µL of *clb* forward primer *(clbA*-F, CAG ATA CAC AGA TAC CAT TCA), 1.0 µL of *clb* reverse primer *(clbA*-R, CTA GAT TAT CCG TGG CGA TTC), DNA template (2.0 µL), and distilled water (4 µL). The amplification reaction was carried out at initialization at 94 °C for 15 minutes, followed by 30 cycles, including denaturation at 95 °C for 30 seconds, annealing at 60 °C for 30 seconds, and elongation at 72 °C for 90 seconds, with final elongation at 72 °C for 10 minutes in a thermocycler (T100 Thermal Cycler; Bio-Rad). Amplicons were electrophoresed in 1.5% agarose agar for 30 minutes at a current of 100 V and examined under ultraviolet light.

### Data Analysis

Data from distributed questionnaires were analyzed and calculated for 95% CI and odd ratio for significant differences (*P* < .05) in estimates of the demographic status, dietary pattern, antibiotic use, alcohol consumption, and hygiene practices as independent variables. The hierarchical clustering of β-lactamase+/*E coli* was evaluated with a heat map that showed the strain antibiotic susceptibility and virulence factor production defining different clusters based on the Euclidean distances from the dendrogram construct generated from the UPGMA evaluation. The significance of β-lactamase–producing *E coli* strains and fecal carriage rates of clb+/EC^bl+^, clb–/EC^bl+^, clb+/EC^bl–^, and clb–/EC^bl–^ was determined by a *t* test and analysis of variance, while the rates of clb+/EC^bl+^ and clb–/EC^bl+^ were compared with a chi-square test (*P* < .05). Independent variables—antibiotic susceptibility pattern, virulence factors (lipase, protease, and hemolysin), biofilm formation, and β-lactamase production—were analyzed with multivariate logistic regression (SPSS version 20; IBM) to correlate and predict the significance of clb expression among the isolates. Multivariate and biplot ordination in principal component analysis was applied in the PAST 4.03 version to investigate the association between the clb+/*E coli* and (1) correlates of the dietary and handwashing hygiene, alcohol consumption, gender, marital status, and ailment variables; (2) antibiotic resistance; and (3) biofilm and virulence factor production. Eigenvalues >1.00 were accounted for the number of selected principal components according to the Kaiser criterion [35]. Components 1 and 2 were extracted for the correlates to provide the possible and significant association of clb carriage with other correlates shown by spatial visualization of the data distribution.

## RESULTS

### Dietary Patterns, Antibiotics, and Handwashing Practice

**Figure 1.**
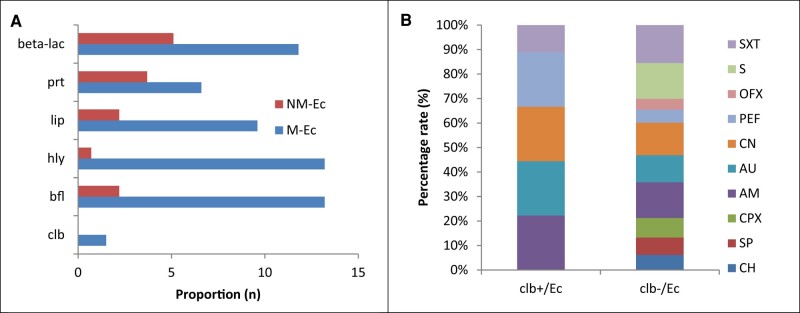
*A*, Distribution of mutant and nonmutant *Escherichia coli* (M-Ec and NM-Ec) encoding colibactin and production of virulence factors. *B*, Antibiotic resistance rates among clb+*/E coli* and clb–/*E coli* (clb+/Ec and clb–/Ec). AM, amoxicillin; AU, Augmentin (amoxicillin and clavulanate); bfl, biofilm; blm, β-lactamase; CH, chloramphenicol; clb, colibactin; CN, gentamicin; CPX, ciprofloxacin; hly, hemolysin; lip, lipase; OFX, ofloxacin; PEF, pefloxacin; prt, protease; S, streptomycin; SP, sparfloxacin; SXT, trimethoprim/sulfamethoxazole.

Of all the *E coli* (N = 136) obtained from the respondents, a lower rate of clb+/*E coli* (1.5%) and higher rates of biofilm (13.2%), hemolysin (13.2%), lipase (9.6%), protease (6.6%), and β-lactamase (11.8%) producers were recorded among the mutant *E coli* strains as compared with the nonmutant types (Figure 1*A*). All the clb+/*E coli* showed resistance to amoxicillin, Augmentin, gentamicin, and trimethoprim/sulfamethoxazole (SXT; Figure 1*B*). Among the 136 respondents whose fecal samples were collected, clb-encoded *E coli* did not significantly differ by demographics: gender (41.9% males vs 58.2% females, *P* = .218), marital status (98.5% single vs 1.5% married, *P* = .222), and age (median ± SD, 18.4 ± 2.51 years vs 26.3 ± 6.34 years; *P* = .200). Based on multivariate analysis, there was no significant association between *clb+/E coli* (n = 2) carriage and the following: consumption of fiber-based food (odds ratio [OR], 1.03; 95% CI, 56.74–138.7; *P* = .213), daily fast food (OR, 1.03; 95% CI, 60.19–145.5; *P* = .216), or food condiments of chemical type (OR, 1.06; 95% CI, 58.20–142.2; *P* = .213), as well as the population that did not consume alcohol (OR, 1.27; 95% CI, 61.74–147.1; *P* = .221). Antibiotic consumption (OR, 1.11; 95% CI, 61.29–145.3; *P* = .222) and handwashing frequency (OR, 1.17; 95% CI, 60.19–145.5; *P* = .216) were not significant risk factors for *clb* gene expression in the fecal samples of respondents (Table 1).

**Table 1.** Multivariate Analysis of the Dietary Pattern, Antibiotics, and Handwashing Practice as Risk Factors for *clb* Fecal Carriage

	Multivariable Analysis of Clb+/blm *E coli*		
Variable	Respondents, No. (%)	OR (95% CI)	*P* Value
Demographics			
Gender (N = 136)			
** **Male	57 (41.9)	1.02 (61.19–147.2)	.218
** **Female	79 (58.2)		
Marital status (N = 136)			
** **Single	134 (98.5)	1.01 (62.74–148.7)	.222
** **Married	2 (1.5)		
Age, y (N = 136)			
** **<20^a^	95 (69.8)	1.45 (68.20–146.8)	.200
** **>20^b^	41 (30.1)		
Dietary pattern			
Diet consumed (N = 136)			
** **Fiber-based food	102 (75.0)	1.03 (56.74–138.7)	.213
** **Vegetarian	34 (25.0)		
Fast food purchase (n = 129)			
** **Daily	62 (48.1)		
** **Weekly	17 (13.2)	1.03 (60.19–145.5)	.216
** **Monthly	10 (7.8)		
** **Occasionally	40 (31.0)		
Food condiments (N = 136)			
** **Natural products	91 (66.9)	1.06 (58.20–142.2)	.213
** **Chemical agents	45 (33.1)		
Alcohol consumption (N = 136)			
** **Yes	21 (15.4)	1.27 (61.74–147.1)	.221
** **No	115 (84.5)		
Antibiotics and handwashing practice			
Antibiotics consumption (n = 126)			
** **Daily	5 (4.0)		
** **Weekly	4 (3.2)	1.11 (61.29–145.3)	.222
** **Monthly	14 (11.1)		
** **Occasionally	103 (81.8)		
Reason for consumption (n = 105)			
** **Abdominal cramps	20 (19.1)		
** **Infections	59 (56.2)	0.87 (50.90–122.2)	.218
** **Diarrhea	13 (12.4)		
** **Prophylaxis	13 (12.4)		
Handwashing frequency (n = 130)			
** **Regularly	89 (68.5)		
** **Once daily	22 (16.9)	1.17 (60.19–145.5)	.216
** **Twice daily	17 (13.1)		
** **None	2 (1.5)		

Significance was set at *P* < .05.

Abbreviations: clb+/blm *E coli*, colibactin-encoded β-lactamase *Escherichia coli*; OR, odds ratio.

^a^Median, 18.4 (SD, 2.51).

^b^Median, 26.3 (SD, 6.34).

### Cluster Analysis of β-Lactamase–Producing *E coli* Strains

**Figure 2.**
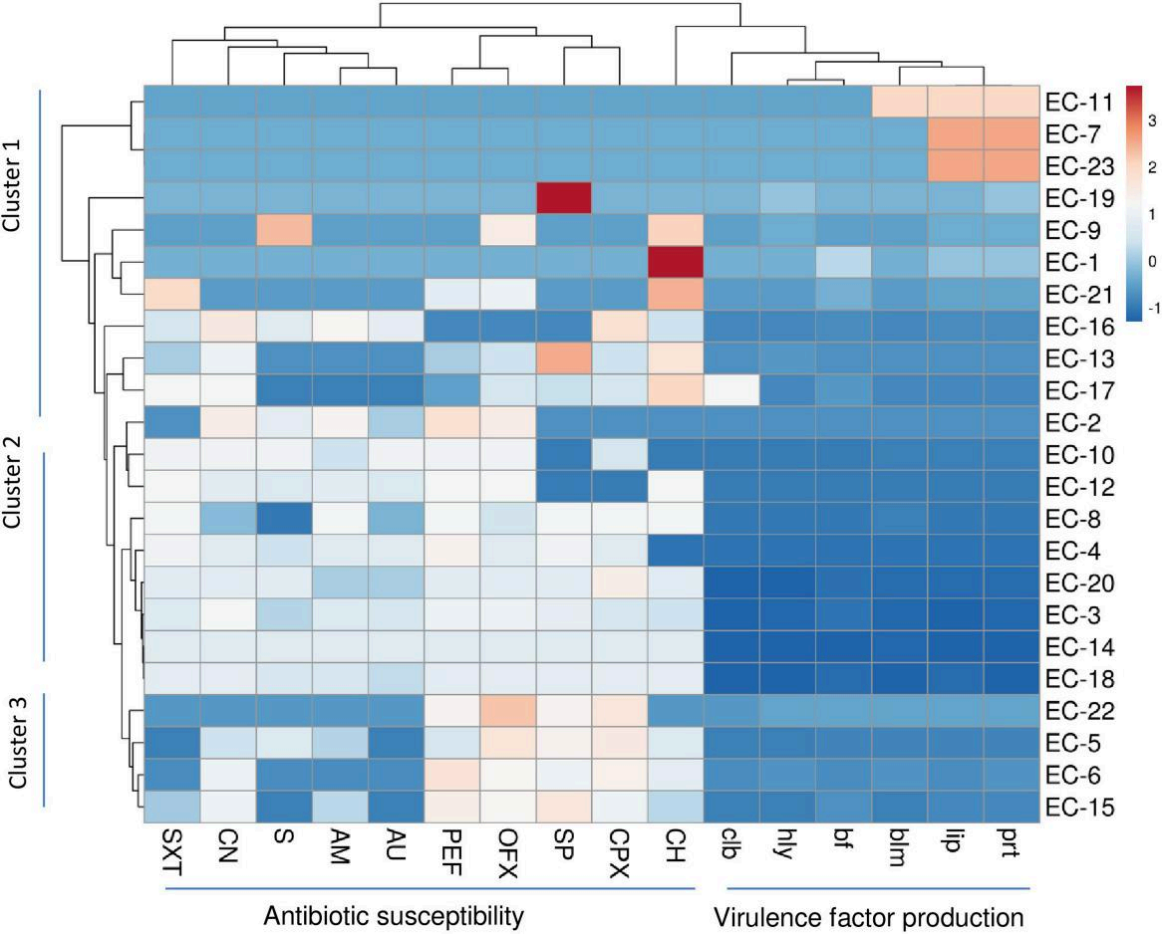
Hierarchical cluster analysis of β-lactamase–producing *Escherichia coli* strains from fecal samples of the students with their antibiotic susceptibility patterns and virulence factor production. AM, amoxicillin; AU, Augmentin (amoxicillin and clavulanate); bf, biofilm; blm, β-lactamase; CH, chloramphenicol; clb, colibactin; CN, gentamicin; CPX, ciprofloxacin; hly, hemolysin; lip, lipase; OFX, ofloxacin; PEF, pefloxacin; prt, protease; S, streptomycin; SP, sparfloxacin; SXT, trimethoprim/sulfamethoxazole.

In Figure 2, all the identified β-lactamase–positive strains were grouped into 3 clusters. High resistance patterns to all the antibiotics, except chloramphenicol and ciprofloxacin, and high production of protease and lipase were observed among strains in cluster 1. Cluster 2 revealed a low susceptibility to >8 antibiotics with very low virulence production. Among the strains in cluster 3, low virulence factor production was recorded with intermediate susceptibility to fluoroquinolones (pefloxacin, ofloxacin, sparfloxacin, and ciprofloxacin) and chloramphenicol.

### Colibactin Detection Among β-Lactamase–Resistant *E coli*

**Figure 3.**
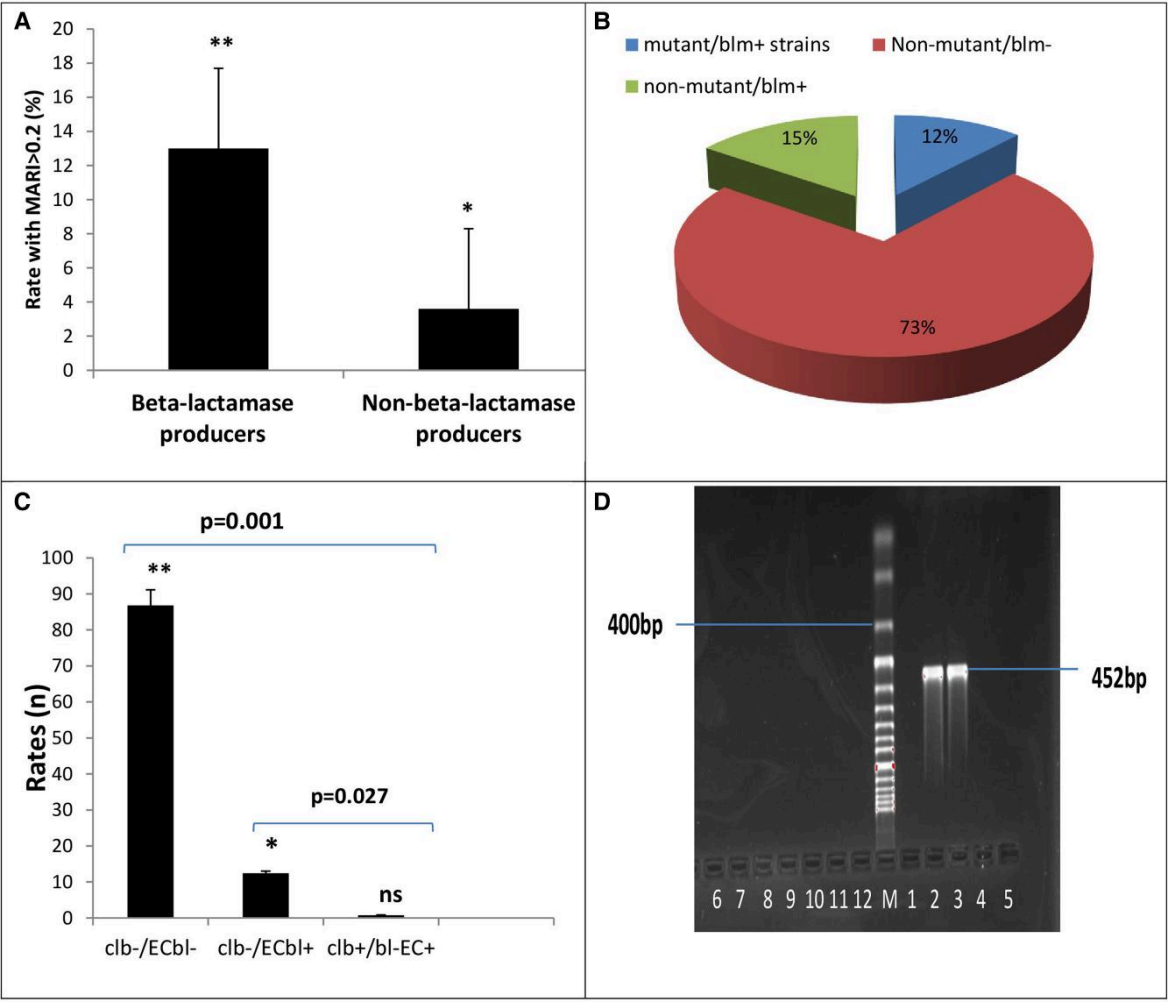
*A*, Proportion of β-lactamase–producing strains with multiantibiotic resistance index (MARI) >0.2. *B*, Distribution of tryptophan mutagenic strains among the β-lactamase (blm) producers. *C*, Detection rate of clb-encoded *Escherichia coli*. bl, β-lactamase; clb, colibactin; EC, *Escherichia coli*; ns, not significant. *A* and *C*, Error bars indicate 95% CI. *P < .01. **P < .05. *D*, Amplicon of *clb* gene from obtained *E coli* (agarose gel 231 electrophoresis of polymerase chain reaction amplicons for the target gene). M, 100- to 1000–base pair DNA molecular weight marker; only lane 6 showed the clb amplicon of 452 base pairs.

A significant rate of 13.2% strains with a multiantibiotic resistance index ≥0.2 was observed among β-lactamase–producing strains (n = 23) vs non–β-lactamase producers, suggesting a high level of resistance to select commonly used antibiotics (Figure 3*A*). A considerable rate of strains (12%) was trp mutant β-lactamase+ while others were nonmutant strains (Figure 3*B*). Only 1.5% of clb+/EC^bl+^ (n = 2) was observed in fecal samples obtained from a participant, which was significantly different from the 12.4% of clb–/EC^bl+^ strains (*P* = .027), while no clb+/EC^bl–^ was recorded (Figure 3*C*). In Figure 2*D*, the agarose gel showed the 452–base pair polymerase chain reaction amplicon band of the *clb* gene from the β-lactamase–producing *E coli*.

### Implication of Identified Variables for clb Carriage

None of the antibiotic variables obtained from the evaluation of phenotypic resistance pattern showed a significant correlation with clb expression (*P* > .05), but only the SXT resistance variables provided a prediction for clb carriage (*P* = .045; 95% CI, .001–.046; Table 2). Virulence factors such as lipase, protease, and hemolysin could not show significant correlations or predictive carriage of clb among the isolates. A positive correlation of biofilm production with clb induction in *E coli* strains was observed (*r* = 0.464, *P* = .026).

**Table 2.** Analysis of Variables With clb Detection

	Correlation Analysis		Regression Analysis				
Variable	*r* Value	*P* Value	β Coefficient	SE	*t* Value	*P* Value	95% CI
Antibiotic							
Chloramphenicol	0.220	.312	−0.770	0.007	−1.891	.101	−.029 to .003
Sparfloxacin	−0.042	.851	−0.646	0.008	−1.200	.269	−.027 to .009
Ciprofloxacin	−0.019	.931	0.397	0.012	0.505	.629	−.022 to .034
Amoxicillin	−0.210	.336	−0.426	0.012	−0.598	.569	−.035 to .021
Augmentin^a^	−0.172	.433	−1.998	0.018	−1.900	.099	−.078 to .009
Gentamicin	0.066	.765	0.818	0.008	1.746	.124	−.005 to .031
Pefloxacin	−0.199	.361	−1.031	0.015	−0.998	.352	−.052 to .021
Ofloxacin	−0.065	.768	0.130	0.018	0.123	.906	−.040 to .045
Streptomycin	−0.197	.368	0.620	0.013	0.772	.465	−.021 to .042
Trimethoprim/sulfamethoxazole	0.097	.659	1.534	0.009	2.438	.045*	.001 to .046
Virulence factor							
Lipase	0.171	.435	−0.345	0.247	−0.584	.578	−.727 to .439
Protease	0.141	.521	−0.828	0.232	−1.583	.157	−.915 to .181
Hemolysin	0.322	.134	0.112	0.165	0.301	.772	−.341 to .440
Other							
Biofilm formation	0.464	.026*	0.533	0.078	1.360	.216	−.079 to .292
β-Lactamase producers	0.243	.264	0.448	0.117	1.575	.159	−.092 to .461

^a^Amoxicillin and clavulanate.

*P < .05.

**Figure 4.**
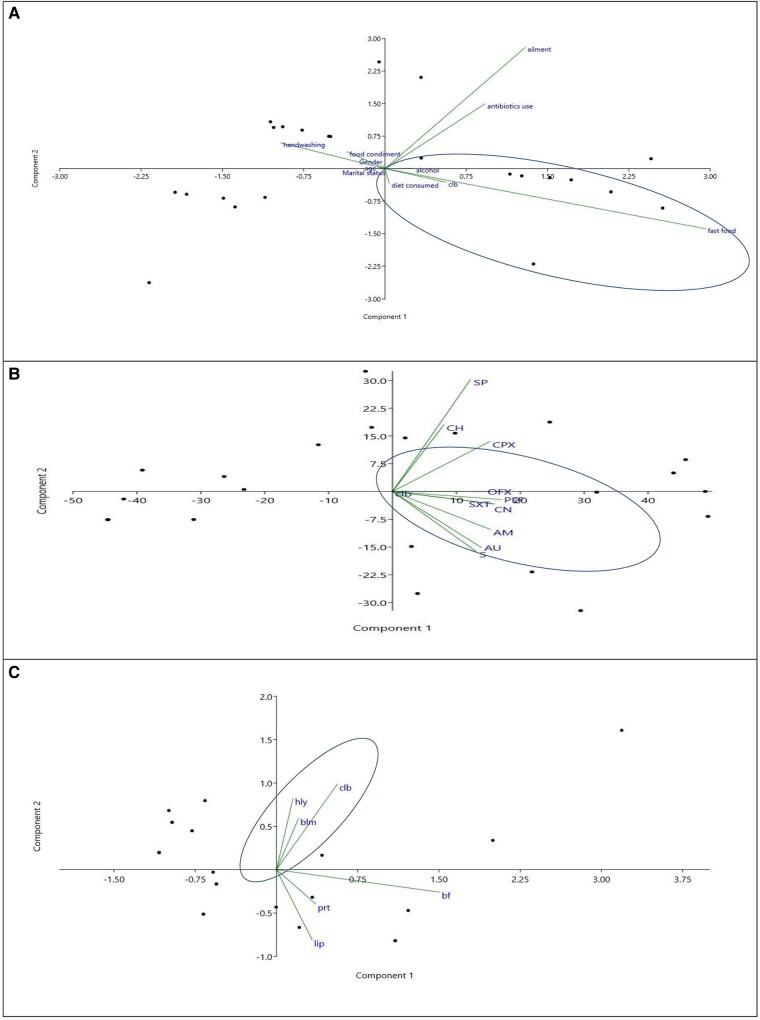
Principal component analysis biplots of β-lactamase *Escherichia coli* isolates: *A*, dietary and handwashing hygiene; *B*, antibiotic resistance pattern; *C*, *clb* carriage, virulence factor production, and antibiotic resistance pattern. AM, amoxicillin; AU, Augmentin (amoxicillin and clavulanate); bf, biofilm; blm, β-lactamase; CH, chloramphenicol; clb, colibactin; CN, gentamicin; CPX, ciprofloxacin; hly, hemolysin; lip, lipase; OFX, ofloxacin; PEF, pefloxacin; prt, protease; S, streptomycin; SP, sparfloxacin; SXT, trimethoprim/sulfamethoxazole.

To explore the implication of dietary patterns, handwashing practice, antibiotic resistance patterns, and virulence factor production for possible induction of the *clb* gene among the *E coli* strains, principal component analysis was utilized. The clb-encoded strains were grouped with the variables for the consumption of fast food and alcohol (Figure 4*A*) and the resistance pattern of SXT, gentamicin, streptomycin, amoxicillin, Augmentin, pefloxacin, and ofloxacin (Figure 4*B*). To investigate the contribution of the produced virulence factors on the expression of clb, the score plot further showed the collection of variables of β-lactamase and biofilm production with clb-encoded *E coli* (Figure 4*C*, Table 2).

## DISCUSSION

The present study investigated the correlates for fecal carriage of colibactin-encoded *E coli* among the student populace, which could trigger the induction of CRC. Considering the confounding factors, the gender, marital status, and age of the respondents were not risk factors for the carriage of the *clb* gene. There was no observed association between fecal clb/*E coli* and consumption of fiber-based food, fast food, or chemical condiment–fortified food or alcohol intake. A fiber-enriched diet among the African populace was reported to reduce the risk of CRC and adenoma [8] as a result of the fiber adsorbing the potential fecal carcinogens, altering bile acid metabolism, and thereby reducing the colonic transit time and pH and increasing production of short-chain fatty acids [36]. Several studies could not confirm the role of alcohol in *clb* in fecal *E coli*, but aging of the gut and persistent consumption of alcoholic beverages could predispose to CRC induction [3, 37]. Antibiotics alter the gut microbiota population and aid colonization of resistance types, which could enhance the risk for the *clb* gene. The burden of antibiotic resistance caused by a high consumption rate as prophylactics and misuse for intestinal infections supports the evidence of oral antibiotics changing the gut microbiota composition and altering essential host immune responses, possibly leading to development and exchange of resistance genes among the gut microbiota [37, 38]. In contrast, long-term antibiotic use in early to middle adulthood could be associated with an increased risk of colorectal adenoma [39]. The burden of intestinal infection would continue to be a leading cause of gut morbidity, which is disproportionately higher among the student populace due to multiple etiologic agents. Improved hand hygiene practice is considered a cost-effective intervention needed to reduce the burden of intestinal diseases, and it would reduce the possible transfer of fecal *clb*/*E coli* [40].

The continuous dissemination of fecal β-lactamase–producing *E coli* is a public health challenge, driving increasing resistance to commonly used penicillin and cephalosporin antibiotic classes. Phenotypic detection of β-lactamase in clustered fecal *E coli* strains showing extended resistance to chloramphenicol and ciprofloxacin suggests indiscriminate use of these antibiotics, which was evident from the high occasional use of antibiotics among the student populace (Table 1). The produced β-lactamase enzymes would further enhance high-level efflux pump activity, alter the drug target (modification of penicillin-binding proteins), and decrease membrane permeability [41, 42]. A separate clustering of β-lactamase *E coli*–producing lipase and protease with low susceptibility to fluoroquinolones and chloramphenicol would describe an imminent pool of resistance enteric strains. This suggests impending intestinal infection severity through stages of tissue damage that involve invasion and necrotization of intestinal epithelia mucosa, leading to gut inflammation and colitis [43].

In addition, the recorded multiantibiotic resistance index ≥0.2 calls for the extension of antibiotic stewardship in tertiary institutions to forestall the spread of antibiotic resistance originating from high-risk fecal sources that could contaminate the environment, particularly the water bodies. To influence or initiate carcinogenesis, detected trp mutant *E coli* could trigger the production of intestinal inflammatory signaling molecules (including interferon γ and interleukin 4), capable of inducing the expression of IDO1 and altering trp metabolism [33, 44], leading to production of high-level indoles from trp metabolism. The produced indole from trp metabolism via the indolic pathway is a key step in inflammatory conditions in colon carcinogenesis, as reported in the fecal samples of individuals with CRC [44].

Recording a low proportion of β-lactamase–producing *E coli* strain encoded with a 452–base pair *clb* gene in the fecal sample of a participant provides indication for possible onset of early CRC. The carriage of clb in the fecal sample of apparently healthy individuals possibly initiates colibactin, acting as a cyclomodulin and causing alteration of the eukaryotic cell cycle, leading to successive enlargement of the nucleus and eventual cell death [45]. Not only could the cytopathic effect of clb mediate cell death by *clb*+/EC^bl+^ strains, but direct contact of the strain with the host cell could necessitate dysfunctional metabolic activity, giving rise to microbial gene products that affect intestinal homeostasis and gut barrier function and leading to microbial dysbiosis, which is commonly found in inflammatory bowel disease and CRC [12, 46]. To date, the epidemiology and prevalence of colibactin in fecal *E coli* are yet to be explored, including their association with intestinal infections in student populations from various geographic locations [12, 47, 48]. To better understand the transmission and prevention of fecal *clb*/*E coli*, future investigation must include the genetic role of associated virulence factors (lipase and protease), β-lactamase enzymes, and risk factor assessment.

Since SXT is one of the commonly used prophylactics for intestinal and extraintestinal infections in Nigeria as compared with other countries [49], the present study revealed the association of SXT resistance with *clb* genes. The SXT-resistant mutant would become dominant and survive through the antibiotic selective pressure as mutant strains. The use of SXT to treat intestinal *E coli* infection has occasionally enhanced the development of high-level resistance to trimethoprim in enterobacteria populations due to changes in cell permeability, loss of drug-binding capacity, and alterations in dihydrofolate reductase, which are mediated by mutation of the chromosomal *DHFR* gene (*dfrA* and *dfrB*) [50]. Persistent DHFR mutants in gut microbiota could further enhance mediation of the *clb* gene in the pks island [50]. The significant association of biofilm formation with fecal clb expression could promote the protective mucosal barrier for effective interaction of the host epithelium, with *clb*+/*E coli* facilitating colonization and invasion of the mucosa epithelial cells [51]. In the tumor microenvironment, as reported in biopsies from patients with CRC, there is high biofilm production by mucosal-invasive *E coli*, showing significant binding affinity to mucus-secreting cells [45]. In vitro, biofilm formation is evidence of cell-to-cell contact, which is important for the genotoxic activity of colibactin, thereby inducing DNA damage that exacerbates mucosal disruption [45, 51–53].

The clustering of the clb-encoded strains with the consumption of fast food and alcohol as variables further affirms the role of poor dietary patterns and alcohol in induction of the *clb* gene. The implication of the β-lactamase production and biofilm aggregation in clb-encoded *E coli* is to enhance the strain intestinal morbidity through invasion and protection from the host immunity and effect of antibiotics, thereby complicating clb expression in the gut mucosa. Further investigations are needed to understand the impact of alcohol on the combined activity of β-lactamase enzymes and biofilm in fecal *clb* expression in healthy individuals.

The limitations of this present study include inadequate provision of demographic data and the limited number of eligible participants providing consent to participate in the study. The inability to collect fecal samples in replicate to aid adequate representation limits the rates of the detected colibactin gene.

## CONCLUSION

The respondents’ demography, dietary, antibiotic, and hand hygiene patterns are not conclusive variables as risk factors for fecal *clb* induction. The association of SXT with clb induction further suggests the antibiotic possible mediation of genotoxic colibactin in the *E coli* pks island. The use of SXT for clinical management or as prophylactics for intestinal infection needs to be controlled. The combined activity of β-lactamase enzyme and biofilm production in the gut microenvironment could promote induction of *clb* in gut *E coli*, thereby facilitating genotoxin production serving as a risk for CRC. Therefore, subsequent genomic studies would provide insight into the colibactin structural mode of induction associated with β-lactamase activity and pks regulation in healthy gut and the physiologic influence in CRC induction.

## Notes

***Acknowledgments.*** We kindly appreciate the staff of the Department of Biological Sciences, Covenant University, Ota, Nigeria, for the collection and storage of the bacterial isolates.

***Authors contributions.*** P. A. A. : conceptualization, methodology, data collection, sample analysis, data analysis, validation, data curation, writing–initial draft, writing–revisions, student supervision, project leadership, project management, and funding acquisition. O. O. I. and O. A. A.: source for the samples, culture and susceptibility analysis. P. A. A. , O. A. A., E. O. A.: data collection, sample analysis, data analysis, validation, data curation. P. A. A., E. O. A. conceptualization, methodology, data collection, sample analysis, data analysis, validation, data curation, writing–initial draft, writing–revisions, student supervision, project leadership, project management. All authors read and approved the final manuscript.

***Availability of data and materials.*** All data generated or analyzed during this study are included in this article.

***Disclaimer.*** CUCRID had no role in study design, data collection and analysis, interpretation of data, the decision to publish, or preparation of the manuscript.

***Patient consent statement.*** Each patient's written consent was obtained before the commencement of the study, and ethical approval for the study was obtained from the Covenant Health Research Committee, Covenant University, Ota, Nigeria (NHREC/CU-HREC/11/04/2023).

***Financial support.*** Small fund was provided by the Covenant University Centre for Research, Innovation and Discovery (CUCRID), Covenant University, Ota Nigeria; to support the collection of the samples for this study.
